# Remarks on the Death of Academicus's Child

**Published:** 1805-07-01

**Authors:** George Custance

**Affiliations:** Kidderminster


### Medical and Physical Journal.

VOL. XIV.] JULY 1, 1805. [NO. LXXVII.

*Printed for R. PHILLIPS, by W. Thorne, Red Lion Court, Fleet Street, London.*

### Remarks on the Death of Academicus's Child.

*To the Editors of the Medical and Physical Journal.*

Gentlemen,

THE second letter of Academicus, respecting the case of his child, inserted p. 516, vol. xiii. of your useful work, excited a considerable degree of interest in my mind, and led me to peruse his former letter, with Mr. Cuming's and Mr. Millett's, with closer attention than I formerly had done.

Upon a review of the whole case, I can see nothing to censure in the conduct and practice of B. D.; and if I did, I should not be satisfied, that I was invested with authority to condemn him.

Mr. Cuming appears to be nearly, though not altogether of my opinion, when he says, "Though delicacy *almost* forbids me to animadvert on the means adopted for the recovery of the child, yet a sense of duty imperiously stifles inferior considerations." One cannot but admire the firmness of Mr. C. when we see him, amidst the conflict of contending passions in his mind, giving the palm of Victory to *Duty*, instead of feebly resigning it into the hands of modest Delicacy. How happy would it be for individuals and the nation, if every public man would sacrifice his feelings to a *proper* "sense of duty." Mr. C. has not, however, favoured the public with a copy of the commission, which imposed upon him such an imperious duty; and therefore I am still at a loss to conjecture, what power delegated to him such authority.

Taking the statement of Academicus as perfectly correct; Mr. C. goes on to determine, ex cathedra, the cause of the disease, and then, notwithstanding the agreement of the physician and apothecary who attended the child, indirectly condemns their practice, and very modestly proposes his own.

It appears, on the second day of the disease, the child had so much debility, that Academicus says, "his sleep was seemingly comatose," and yet "in such cases as these" Mr. C. informs us, "he has often had recourse to the warm bath, keeping the child in it until it became faint." But it may still be doubted, whether this plan would have succeeded better than that which was pursued.

Notwithstanding Mr. C. seems very *certain* that obstructed perspiration was the sole cause of the disease, it is not quite clear that worms in the alimentary canal may not have occasioned both the fever and green stools.

Does not also the "moaning for hours," on the night after the attack, naturally suggest, at least, the possibility that there may have been some affection of the brain, which terminated in hydrocephalus internus; especially when we consider, that the coma increased, and repeated convulsions took place, before the dissolution of the child? Indeed, Mr. Cuming himself is of opinion, that "the fatal symptoms of coma and convulsions, which closed the scene of this child's illness, might have been occasioned by hydrocephalus." If that was the fact, the possibility is, that inflammation of the brain very early supervened, if it was not the primary disease.

Mr. C. very properly observes, that the "connection which subsists between the bowels and the skin is very striking; when the pores are obstructed, diarrhœa or fever, for the most part supervenes, and is frequently attended with sickness or vomiting." In this case, however, we are not informed that either sickness or vomiting took place. It may, therefore, reasonably be doubted, whether obstructed perspiration was the cause of the disease, and whether an emetic and sudorifics would have been more beneficial than the medicines exhibited by B. D.

I like the reasoning of B. D. upon this point very much. He tell us he gave no emetic. 1st. "Because there was no sickness nor nausea." 2dly. Because Nature had pointed out the path to be pursued." Now, as B. D. was on the spot, and saw the child very early in the disease, he had certainly the best opportunity of noticing the way which Nature took to relieve herself of the injury, however incurred: and being the confidential apothecary of this family, I conclude, that he possessed, even in the judgment of Academicus himself, a competent share of professional ability to decide, both as to the nature of the disorder, and the best method of treating it.

Nothing

Nothing can exceed the good sense with which Mr. Millett, junr. writes on the subject; in his letter (page 51, vol. 12.) the wisdom of an enlightened practitioner and the candour of a gentleman are both very conspicuous, and cannot fail in pleasing every liberal and impartial reader.

It is much to be lamented, that an unsuccessful case should destroy Academicus's good opinion of a "confidential practitioner;" and it is no less painful to observe how very anxious he seems (p. 516 and 517, vol. xiii.) to criminate the conduct of B. D. and to impeach his judgment.

Upon the whole of the circumstances taken together, allow me to make a few observations.

1st. It is very desirable, in all doubtful cases, to have the body examined soon after death. Had this child been opened, something might have been discovered that might have satisfied the mind of Academicus, that *even an emetic* could not have saved his life.

2dly. It is very unreasonable to judge of a practitioner's skill by the fatal termination of a disease. "Medical men are certainly the instruments towards the recovery of people from sickness;" but it should always be remembered, that they are *only* instruments. Diseases can be removed by him alone, who inflicts them. But as he employs instruments in bringing on any disease, so also he makes use of them in taking them away. Medical men cannot be expected to work miracles, and therefore should not hastily be blamed when their exertions are not attended with the desired success. But as, without means, we are not warranted in expecting a divine interposition; it is our duty to use such as human wisdom and experience justify us in employing. But depending solely upon the means we use, without imploring the divine blessing, is presumption: So that when one hears another say, (page 568, vol. xiii.) "Except in cases of a natural or artificial old age, or, in other words, when the constitution has been exhausted either by intemperance or by time, no person *ought* to die of Fever;" one naturally believes, that as "dead flies cause the ointment of the apothecary to send forth a stinking savour, so doth a *little folly* him that is in reputation for wisdom and honour."

3dly. The knowledge of medicine, possessed by persons out of the profession, generally does them more harm than good. A gentleman may certainly read on medical subjects, till he understands much in theory; and yet not be able to apply his knowledge to one good practical purpose. Just as he may, by reading Blackstone's Commentaries, thoroughly understand the British constitution, and yet make a very bad lawyer or statesman. Practice alone can produce that sagacity in discriminating, and that decision in treating diseases, which are so essential to insure success. Every practitioner remembers, notwithstanding the stock of theoretic knowledge he brought with him from the University and Hospitals, what doubts and fears have filled his mind when first called, in private practice, to the sick beds of his valued friends or endeared relatives; and what wavering and indeterminate conduct characterized the treatment of his early patients. How then can gentlemen, who, like Academicus, study medicine as an accomplishment, be competent to decide on the nature of diseases, or the best mode of treating them? Affection will alarm where there is no danger; and the want of sagacity and promptness will often render the disorder fatal. Such persons should always employ a "confidential practitioner," and suffer his own judgment to be subordinate to his friend's. Most medical men are so aware of the propriety of this conduct, that they usually commit themselves and families, when sick, to the judgment and care of their brethren in the Faculty.

Lastly, It is our duty, when we are bereaved of our dearest relatives, to bear our loss with becoming submission to the Divine Will. The propensity there generally is in survivors to blame the medical attendants, when their friends or relations die, is both ungrateful and sinful. The largest sum of money (generally small enough) is a poor compensation to a man of a liberal education and refined feelings; when his assiduity, time, council, patience, and kindness, are returned with the most stinging and mortifying reflections; merely, because the event, which he could not controul, has been contrary to his warmest wishes. Surely, gratitude is due to a professional man, whatever be his success, unless he can be fairly charged with wilful neglect, or *indisputable* and gross mistakes.

This propensity to condemn the conduct of medical men is sinful, inasmuch, as it discovers a murmuring and discontented spirit; and is, in effect, if not in words, "charging God foolishly."

Academicus, probably, is a clergyman; if not, he is doubtless a gentleman of education and refinement. Whilst, therefore, I most tenderly sympathize with him, on the loss of a beloved child, I cannot but hope, that his good good sense will induce him, upon mature reflection, to repose again in Mr. B. D. the confidence his family formerly placed in him. He will also, it is hoped, not perplex his mind, with imaginary cases and modes of cure, by reading medical books on the diseases of children; but will charitably conclude that the physician and Mr. B. D. did all that his child's case required, or that human means could effect. I am, &c.

*Kidderminster, June* 17, 1805. GEORGE CUSTANCE.

